# Status epilepticus: the score question

**DOI:** 10.3389/fneur.2025.1721546

**Published:** 2025-12-09

**Authors:** Francesco Misirocchi, Pia De Stefano

**Affiliations:** 1 Unit of Neurology, Azienda Ospedaliero Universitaria di Parma (AOSP), Parma, Italy; 2 Neuro-Intensive Care Unit, Department of Intensive Care, University Hospital of Geneva, Geneva, Switzerland; 3 Department of Clinical Neuroscience, University of Geneva, Geneva, Switzerland

**Keywords:** status epilepticus, prognostic models, risk prediction, outcome assessment, artificial intelligence

## Abstract

Status epilepticus (SE) is a heterogeneous neurological emergency associated with high morbidity and mortality. Multiple prognostic scores have been developed, most of which primarily focus on short-term mortality. The Status Epilepticus Severity Score (STESS) and the Epidemiology-Based Mortality Score in SE (EMSE) remain the most extensively validated tools, while more recent models have addressed specific subgroups, functional outcomes, long-term survival, epilepsy development, and recurrence risk. Despite these advances, the predictive performance of current scores remains limited, largely due to reliance on post-hoc or subjective variables, lack of universally applicable cut-offs, and limited external validity. Moreover, their integration into clinical algorithms is minimal, confining their use mainly to retrospective risk adjustment in research context. Emerging approaches based on artificial intelligence offer improved predictive performance, but their clinical applicability is hampered by several issues. Future SE models should balance feasibility with accuracy, integrate variables across different stages of SE care, and align with standardized SE definitions and management, to become clinically valuable tools able to guide individualized SE management.

## Introduction

Status epilepticus (SE) is a life-threatening neurological emergency defined by prolonged or recurrent seizures resulting from failure of seizure-termination mechanisms or processes that provoke abnormally sustained seizures (1). SE is highly heterogeneous in both clinical presentation and etiology, encompassing acute symptomatic, chronic, and progressive conditions. It can occur at any age and in patients with diverse comorbidities, complicating prognostication and therapeutic decision-making (2). This review critically examines prognostic scores developed for SE, evaluates their clinical utility and limitations, and outlines the essential features of an ideal risk-stratification model.

## The clinical challenge of stratifying patients with SE

When faced with a patient with suspected SE, clinicians must rapidly address several critical questions, like how this patient should be treated, who and where should the patient be managed [Intensive Care Unit (ICU) or not], how the patient should be monitored [continuous EEG (cEEG) vs. spot EEG], is SE the primary driver of deterioration or a secondary phenomenon, what is the risk of death or permanent disability from this episode, what is the likelihood of future epilepsy or SE recurrence.

Despite the centrality of these questions in acute care, only some are addressed by current guidelines or existing scoring systems (3). In many cases, decisions are driven primarily by clinical judgment, institutional resources, and physician experience (4–7).

This underscores the need for tools that enable timely, evidence-based decisions about treatment strategies, monitoring priorities, and family counselling (8). Although numerous prognostic scores have been proposed in recent years, mostly targeting in-hospital mortality (3), their real-world utility remains limited, particularly in guiding therapeutic escalation, predicting functional outcomes, and assessing long-term seizure risk.

## The rise of scores in SE

Efforts to reduce uncertainty and support clinical decision-making in SE have led to the development of numerous scoring systems, which are also widely used in epidemiological studies and clinical trial design. Most of these tools aim to predict in-hospital mortality or survival based on combinations of anamnestic, clinical, radiological, laboratory, and EEG parameters. Table 1 provides an overview of all published SE scoring systems, detailing the main variables included, the size and characteristics of the derivation cohorts, and the presence of internal and/or external validation.

**Table 1:** Main prognostic scores proposed for status epilepticus (SE).

Score	Original article	Population	Cohort size	Variables assessed	Suggested cut-off	Primary endpoint	Internal validation	External validation
ACD score	Roberg LE et al., JAMA Neurology, 2022	Adults; non-hypoxic SE;	261	Age, level of consciousness, duration of SE	No cut-off	2-year mortality	Yes	Yes
AFTER score	Rodrigo-Gisbert M et al., Epilepsy Behav, 2023	Adults; SE with acute symptomatic and cryptogenic etiologies	230	Cryptogenic etiology, first-line treatment initiation ≥1 h, EEG RPPs, super-refractoriness	≥2	5-year seizure recurrence/epilepsy	No	No
EMSE	Leitinger M et al., Neurocritical Care, 2015	Adults; all SE	92	SE Etiology, age, comorbidities, EEG features (+ SE duration, level of consciousness)	≥64	In-hospital mortality	No	Yes
END-IT score	Gao Q et al., Crit Care, 2016	> 12 years old; non-hypoxic CSE	132	Encephalitis, NCSE, diazepam resistance, imaging abnormalities, tracheal intubation	≥3	Unfavorable functional outcome at 3 months (mRS 3–6)	No	Yes
M3A2S2H	Almufti et al., Journal of Intensive Care Medicine, 2025	Adults; all CSE	123′082	cardiac arrest, age, acute symptomatic CSE, mechanical ventilation, sepsis, metastases, chronic liver failure, medication nonadherence	No cut-off	In-hospital mortality	No	No
PEDSS	Tiwari et al., Epilepsia, 2020	<14 years old; all CSE	61	PCPCS, background EEG, drug refractoriness, seizure semiology, presence of critical sickness	≥3, ≥4	In-hospital PCPCS, 3-month PCPCS	No	Yes
SACE score	Misirocchi F et al., Epilepsia, 2024	Adults; non-hypoxic NCSE	116	Salzburg EEG criterion A2, age, history of seizures, coma	≥3	In-hospital survival	No	Yes
SEDS score	Damien C et al., Seizure, 2025	Adults; non-hypoxic RSE	54	Daily SOFA cardiac and respiratory dysfunction variables, CRP, GCS, EEG RPPs, continuous IV anesthetic use	No cut-off	Unfavorable in-hospital functional outcome (mRS 4–6)	Yes	No
SEM score	Sirikarn P et al., BMC Neurology, 2019	All SE	13′910	Age, gender, brain tumor, stroke, cancer, diabetes, chronic kidney disease, pneumonia, urinary tract infection	No cut-off	Long-term (1–10 years) mortality	Yes	No
STEPSS	Sidharth et al., Seizure, 2019	<18 years old; all CSE	140	consciousness, history of previous seizures, worst seizure type and age	≥4	In-hospital mortality, POPC, treatment response	No	Yes
STESS	Rossetti AO et al., Neurology, 2006	Adults; non-hypoxic SE	107	Age, worst seizure type, level of consciousness, history of previous seizures	≥3, ≥4	In-hospital mortality	Yes	Yes
mSTESS	González-Cuevas M et al., Eur J Neurol, 2016	Adults; non-hypoxic SE	136	STESS variables + baseline mRS; age cutoff modified	≥4	In-hospital mortality	No	Yes
nSTESS	Huang TH et al., Healthcare 2021	Adults; non-hypoxic SE	59	STESS variables + barbiturates + number of ASMs within first week	≥4	In-hospital mortality	No	Yes
A-STESS	Misirocchi F et al., Eur J Neurol, 2025	Adults; non-hypoxic SE	496	STESS variables (replacing age with serum albumin)	≥3	In-hospital mortality, 6-month mortality	No	No
STESS+END-IT	Jiang Y et al., Epilepsy Behav, 2020	Adults; NCSE or generalized non-hypoxic CSE	123	Combination of STESS and END-IT items	STESS ≥ 4, ENDIT ≥ 3	In-hospital mortality	No	No
Thailand risk score	Tiamkao S et al., Epilepsia, 2018	All SE	10′ 924	Age, 5 SE comorbidities, 5 SE complications	≥4	30 days mortality	Yes	Yes

For each score, main variables included, the size and characteristics of the derivation cohorts, primary outcome, suggested cut-off (when available), and the presence of internal and/or external validation. SE, status epilepticus; mRS, modified Rankin Scale; ASMs, antiseizure medications; CSE, convulsive SE; NCSE, non-convulsive SE; RSE, refractory SE; PCPCS, Pediatric Cerebral Performance Category Scale.

The Status Epilepticus Severity Score (STESS), originally designed to estimate in-hospital mortality in adults with non-hypoxic SE, remains the most widely used score due to its simplicity (9, 10). It includes four variables: level of consciousness, worst seizure type, age (>65 years), and history of previous seizures. The initial cut-off was ≥3 (STESS-3), indicating higher mortality risk. A subsequent external validation suggested raising the threshold to 4 (STESS-4) to improve its low positive predictive value (PPV) (11), but no cut-off achieved both high sensitivity and specificity (8). Although STESS is the most extensively validated SE score, several refinements have been proposed to enhance predictive accuracy:

1. Modified STESS (mSTESS), which integrates the baseline modified Rankin Scale (mRS) and raises the cutoff age from 65 to 70 years (12);
2. New modified STESS (nSTESS), which incorporates the use of barbiturates and the number of antiseizure medications (ASMs) administered within the first week (although limiting its utility as an early prognostic tool) (13, 14);
3. Albumin-STESS (A-STESS), which replaces age with serum albumin as a more reliable surrogate of the patient’s patho-physiological status (15).

Another well-known score is the Epidemiology-Based Mortality Score in Status Epilepticus (EMSE), developed to predict in-hospital mortality by converting published mortality rates for specific predictors into a scoring system (16). EMSE applies to adults, including those with hypoxic–ischemic etiologies, and integrates six parameters: age (A), etiology (E), duration (D), EEG findings (E), comorbidities (C) and level of consciousness before treatment (L), with a cut-off of 64 points identifying higher mortality risk. Shorter versions of EMSE with different combinations of these parameters exist, the leading ones being EMSE-EACE and EMSE-EAC, because some of its features may not always be available. In its original study, EMSE outperformed both STESS-3 and STESS-4, but its superiority was not consistently observed across all cohorts (8). Moreover, EMSE requires clinical data that may not be available at the time of SE onset and, like STESS, more accurately identifies patients who are likely to survive SE, while its ability to predict mortality remains less reliable. Recently, a nomogram based on EMSE parameters demonstrated better predictive accuracy for 30-day mortality than EMSE alone (17).

Both STESS and EMSE have been evaluated for functional outcome prediction using mRS or Glasgow Outcome Scale (GOS), with inconsistent results (18, 19). To address this gap, the *END-IT* score [Encephalitis, Nonconvulsive SE (NCSE), Diazepam resistance, Imaging abnormalities, Tracheal intubation] was proposed in 2016 to predict unfavorable functional outcome at 6 months after convulsive SE (CSE) (20). END-IT includes diazepam resistance, neuroimaging abnormalities, and intubation, alongside variables already considered in prior scores. Patients with scores ≥3 had worse outcomes. However, its binary categorization of “encephalitis vs. not” contributes less prognostic value in older patients with different etiologies. Whether reclassifying or expanding etiology categories would improve performance remains unclear. Because STESS and END-IT incorporate different predictors, a combined *STESS+END-IT score* was subsequently tested, demonstrating improved accuracy for in-hospital mortality prediction compared with either score alone (21).

A recent systematic review and meta-analysis, including all studies applying STESS, EMSE, and END-IT, as well as their original derivation cohorts, showed that these scores perform suboptimally, particularly in critically ill or refractory SE populations (8). Overall, they demonstrated only acceptable AUC, sensitivity, and specificity, but consistently exhibited very low PPVs, limiting their ability to reliably identify patients at risk of poor outcomes. Among the 21 included studies, 12 had an unclear risk of bias, and 9 had a high overall risk of bias, primarily due to small sample sizes and narrowly selected patient populations (8).

Other adult-focused models include two scores derived from the Thai national SE database (22, 23). The first, predicting in-hospital mortality, incorporated 11 variables (age, five comorbidities, five complications) and identified a cut-off of 4 as associated with 30-day mortality. The second, the Status Epilepticus Mortality (SEM) score, estimated 10-year mortality using demographics (including sex), comorbidities, and complications, with a maximum score of 64 (23). More recently, a simpler tool, the *ACD score* (Age, Consciousness, Duration; maximum 15 points), was developed in Denmark and externally validated in two cohorts (24). ACD correlates linearly with 2-year survival, particularly in etiologies with low risk of structural damage. However, its reliance on SE duration, which can only be determined retrospectively, limits its real-time applicability (8). Another approach combined clinical predictors (refractoriness, SE duration, *de novo* SE) with serum biomarkers (lipids, progranulin) to predict both short- and long-term outcomes in ICU-admitted adult SE patients, although biomarker availability constrains its use (25).

While most scores have been developed in mixed SE populations, a model specifically for NCSE was proposed in an Italian cohort: the *SACE score* (26). It combines the Salzburg EEG criterion A2 with age (≥75 years), seizure history, and level of consciousness. At a cut-off of ≥3, it achieved 77% sensitivity, 74% specificity, and 76% overall accuracy, outperforming other tools for this SE subtype (26, 27).

For refractory SE, the recently introduced SE Daily Severity Score (*SEDS*) (28) monitors ICU patients’ daily severity and predicts discharge outcome (mRS < 4 vs. ≥ 4) using cardiovascular and respiratory SOFA components (29), C-reactive protein, Glasgow Coma Scale, EEG patterns (rhythmic and periodic patterns, ictal-interictal continuum), and continuous intravenous anesthetic use.

To date, only one score has been specifically tailored to evaluate the risk of developing epilepsy after *de novo* status epilepticus (30). The *AFTER* score estimates the 5-year post-SE epilepsy risk using cryptogenic etiology, delayed first-line treatment (≥1 h), rhythmic and periodic EEG patterns, and super-refractoriness, with scores ≥2 indicating a high risk (30). More recently, a machine learning model using the Random Forest algorithm outperformed traditional methods in predicting 2-year seizure recurrence after *de novo* SE (31).

The most recent adult prognostic model, the Status Epilepticus *M3A2S2H* score, was derived from the U.S. National Inpatient Sample database in CSE and incorporates 8 predictors of in-hospital outcome: hypoxic–ischemic encephalopathy, age >60 years, acute symptomatic SE, mechanical ventilation, sepsis, metastases, liver disease, and no medication exposure etiology (32).

In pediatric populations, two scores have been proposed. The Status Epilepticus in Pediatric Patients Severity Score (STEPSS), adapted from STESS, predicts in-hospital mortality, unfavorable neurological outcome, and treatment response using four variables (consciousness, prior seizures, seizure type, age), with a total score of 6 and a cut-off of 3 (33). The *PEDSS* score integrates pre-SE PCPCS (34), EEG abnormalities, drug refractoriness, semiology, and critical illness to predict mortality and discriminate outcomes at discharge and 3 months, with optimal cut-offs of 4 and 3, respectively (35). Both were developed in exclusively CSE cohorts and have undergone external validation (36, 37).

## Limitations of current prognostic scores

The ongoing development of new prognostic scores reflects both the high clinical demand and the unresolved nature of risk stratification in SE. Despite their proliferation, most existing tools suffer from limitations that restrict their usefulness in everyday clinical practice.

First, most scores focus exclusively on short-term mortality, providing limited insight into functional outcomes (28, 38) or long-term prognoses such as epilepsy development and SE recurrence (15, 23, 24, 30). Second, from a statistical standpoint, many scores lack clearly defined, clinically actionable cut-offs, and overall pooled sensitivity and specificity remain suboptimal (3, 8, 11). This limitation also raises concerns about self-fulfilling prophecies: tools designed to predict short-term mortality may inadvertently influence therapeutic decisions, despite having better ability to identify patients with good rather than poor outcome.

Interestingly, despite differences in variables assessed, the pooled performance of STESS, EMSE, and END-IT is broadly similar (18, 20, 26). One explanation may be that their variables, although distinct, are correlated and capture overlapping clinical dimensions (8). Table 2 further illustrates this redundancy: among the 16 identified scores, 11 include age, 13 incorporate anamnestic factors, and 10 rely on assessments of consciousness level or SE semiology. More broadly, many scores depend on subjective or post-hoc variables, such as seizure duration or detailed semiology classification, which are influenced by treatment decisions (e.g., early sedation) and inconsistently documented, thereby undermining reproducibility and external validity (8). In contrast, more objective predictors such as EEG findings, and particularly imaging and laboratory data, remain underrepresented.

**Table 2:** Main feature categories (age, anamnestic features, consciousness, status epilepticus (SE) semeiology, SE etiology, EEG features, imaging features, laboratory features, treatment features and SE duration/ refractoriness) for each score.

Score	Age	Anamnestic features	Consciousness	Semiology	Etiology	EEG	Imaging	Laboratory	Treatment	Duration/refractoriness
ACD score	X	X	X							
AFTER score					X	X			X	X
EMSE	X	X	X		X	X				X
END-IT score				X	X			X	X	
M3A2S2H	X	X		X	X				X	
PEDSS		X								
SACE score	X	X	X			X				
SEDS score			X			X		X	X	
SEM score	X	X			X					
STEPSS	X	X	X	X						
STESS	X	X	X	X						
mSTESS	X	X	X	X						
nSTESS	X	X	X	X					X	
A-STESS		X	X	X				X		
STESS+END-IT	X	X	X	X	X		X	X		
Thailand risk score	X	X			X				X	
Total	11	13	10	8	7	4	1	4	6	2

Crucially, current prognostic tools are not effectively integrated into therapeutic algorithms. They do not provide guidance on practical questions, such as whether a patient should be admitted to the ICU, when to escalate to anesthetic therapy, or how to prioritize cEEG monitoring. Consequently, their role remains passive: they are mainly applied retrospectively for severity adjustment in observational studies rather than to prospectively guide treatment decisions. This limits their clinical relevance and reinforces their academic rather than operational value. As emphasized by Alvarez and Rossetti, the next critical step should be a randomized trial comparing standard management with a strategy based on decision algorithms incorporating prognostic scores, to determine whether these tools can really influence patient outcome (39).

From a broader perspective, several general ICU severity scores, such as SOFA, APACHE II, and SAPS, also apply to patients with SE (29, 40). In intensive care settings, these global severity scales, which assess the patient’s overall physiological burden rather than focusing solely on SE features, often predict SE patients’ outcome as well as, or even better than, SE-specific tools (27, 41). However, these scores are not designed for use at SE onset: they are calculated upon ICU admission and often repeated daily to track individual trajectories. They also rely on variables that are rarely available at the initial clinical evaluation of a suspected SE, which limits their practicality compared with the more immediate, bedside-oriented nature of SE-specific scores.

Finally, among tools for patients at risk of SE, two scores are specifically designed to identify those at higher risk of seizures or evolving SE, who may benefit from prolonged EEG monitoring. The TERSE score (Time-dependent Risk of Seizures in Critically Ill Patients on Continuous EEG) estimates the likelihood of subsequent electrographic seizures by combining clinical features with early EEG patterns, guiding decisions on cEEG duration (42). Similarly, the 2HELPS2B score stratifies seizure risk in acutely ill patients using early EEG findings and seizure history; a score ≥2 indicates that cEEG should be continued for at least 24 h (43).

## The role of artificial intelligence: hype, potential, and limits

Artificial intelligence (AI) and machine learning (ML) have recently emerged as promising tools in prognostication across various fields of medicine, including neurology (44). Their appeal lies in the possibility that algorithms trained on large datasets may uncover hidden patterns and offer individualized predictions beyond the reach of conventional scoring systems (45). Recent studies have applied various AI/ML approaches, such as random forests, support vector machines, and neural networks, to predict outcome in multiple clinical conditions, including SE. Compared with conventional regression-based models, these algorithms can model nonlinear and high-dimensional relationships; however, they are also particularly prone to overfitting, especially when sample sizes are small relative to the number of predictors. Model comparison should consider not only discrimination and calibration metrics but also penalized or Bayesian criteria (AIC, BIC, Bayes factors) that take model complexity into account.

Moreover, despite their potential, several important caveats remain. Many ML systems operate as “black boxes,” generating predictions without transparent or interpretable reasoning (40). Furthermore, model performance critically depends on data quality: retrospective hospital datasets often contain missing or unverified values, inconsistent variable definitions, and substantial variability in treatment practices, all of which impair accuracy and generalizability (44). Finally, AI systems lack contextual awareness. A model may recommend ICU transfer for a high-risk patient without considering bed shortages or the presence of an advance directive limiting invasive interventions, whereas clinicians routinely integrate nuanced contextual factors, such as family dynamics, cultural considerations, subtle clinical cues, that algorithms cannot capture (45). For these reasons, models grounded in interpretable variables and clearer mechanistic plausibility currently remain more suitable for clinical neuroprognostication, unless AI/ML approaches demonstrate reproducible and clinically meaningful improvements in external validation.

## A broader perspective: definitional and practical challenges

Despite advances in predictive modelling, three persistent challenges undermine both the development and real-world implementation of SE prognostic scores:

### The definition of SE

While formal definitions of SE exist (1, 46), such as the 5-min cutoff for CSE and the 10-min threshold for focal or generalized NCSE in adults, applying them in real-world practice is often challenging. Presentations can be ambiguous, vary over time, and be confounded by treatment effects (47). EEG plays a critical role in resolving uncertainty, but its availability differs widely across centers, and interpretations are influenced by sedatives or anesthetics (48). Key diagnostic grey zones include: (1) unknown NCSE onset in patients not under cEEG monitoring, which represents most cases; (2) altered consciousness with motor features that resolve spontaneously or after benzodiazepines without EEG confirmation; (3) apparent seizure cessation after intubation and sedation, with unclear electroclinical correlation; (4) recurrent seizure clusters during the same hospitalization with uncertain inter-episode intervals; and (5) electrographic or electroclinical SE patterns in severely ill patients, such as anoxic SE (49). These ambiguities lead to inconsistent classification, reducing the reliability of prognostic tools.

### Variability in SE treatment guidelines

Although three-step treatment algorithms are well described in guidelines, adherence in practice is inconsistent (7, 50). Deviations in drug selection, timing, and escalation strategies introduce significant heterogeneity in patient outcome (6, 7, 51). This variability directly affects the validity of many prognostic scores. For example, scores such as STESS or END-IT include variables (e.g., level of consciousness, need for intubation) that are heavily influenced by local practice, including thresholds for ICU admission and speed of escalation to anesthetics (10, 20). These elements are heavily influenced by local practice patterns, such as thresholds for ICU transfer, and speed of escalation to anesthetic agents. Moreover, patients may undergo intubation without the concurrent and continuous administration of anaesthetics, for example in cases where they remain in a comatose state during the postictal period, misdiagnosed as a persistent SE. As a result, the same patient may receive different scores in different institutions, which limits the external validity and generalizability.

### Expertise in EEG interpretation

Despite the availability of standardized terminology (52), substantial variability persists in EEG expertise among clinicians managing SE. In critically ill SE patients, cEEG monitoring coupled with timely and accurate interpretation is essential (53). However, specialized neurophysiologists are rarely available around the clock, particularly in smaller centers or during off-hours, creating a gap between guideline recommendations and real-world practice. To address this limitation, recent studies have explored AI-assisted EEG interpretation in ICU settings (54–56). These approaches have shown promising and sometimes remarkable results, suggesting that AI may help enhance diagnostic accuracy and reduce interobserver variability (54–56).

Without a universally applicable, operationalized definition of SE that addresses diagnostic grey zones, and without widespread adoption of standardized EEG terminology and treatment protocols with clearly defined timelines, any rapid clinical score will deliver only limited real-world benefit. This contrasts with stroke care, where prognostic tools gained clinical acceptance through the combination of uniform definitions, strict time windows, and standardized pathways.

## Toward an ideal risk stratification model

An ideal SE scoring system should function not merely as a mortality estimator but as a decision-support tool, guiding individualized treatment and monitoring strategies. It should act as a “compass” for clinicians, helping determine the optimal level of care, the urgency of escalation, and the appropriate intensity of monitoring.

**Figure 1:**
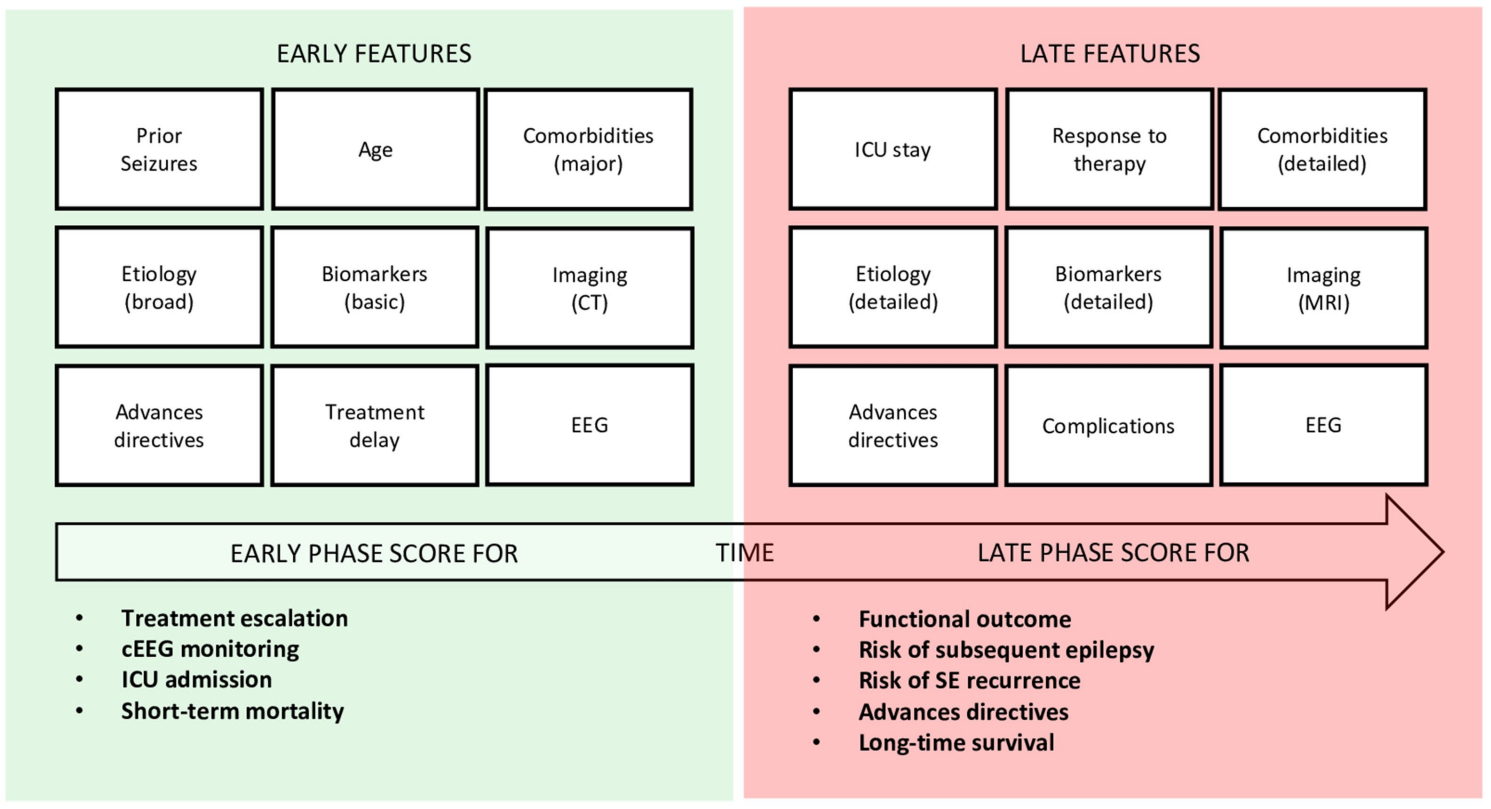
Conceptual two-stage framework for prognostic scoring in status epilepticus (SE). Early-phase models rely on standardized and rapidly accessible variables to support acute management and short-term mortality prediction, whereas late-phase models incorporate dynamic clinical, imaging, and electrophysiological data to estimate functional outcome, long-term survival, and the risk of epilepsy or SE recurrence.

Developing such a tool requires balancing predictive accuracy with feasibility. Predictors should be non-redundant, add independent prognostic value, and be rapidly obtainable in most settings. Subjective or inconsistently assessed variables, such as precise seizure semiology and seizure duration, should be avoided, as they are often confounded by sedation or local practice. A single static score is unlikely to address all clinical scenarios. Instead, a two-stage approach may be more effective (Figure 1):

- Early-stage model (emergency department or ICU admission): designed for rapid decision-making, such as ICU triage, cEEG initiation, and timing of therapeutic escalation.
- Late-stage model (during hospitalization): integrating dynamic information (treatment response, neuroimaging evolution, extended EEG findings) to predict longer-term outcomes, including functional recovery, epilepsy development, and risk of SE recurrence.

SE often occurs along a spectrum. SE may represent either a complication of another underlying condition (e.g., cardiac arrest, stroke, metabolic disturbances), or, at the opposite extreme, the primary driver of the patient’s clinical state (e.g., epileptic syndromes, underdosed antiseizure medications), with many cases falling in between. Prognosis is thus determined by a combination of SE-related and non-SE-related factors. A two-stage model might accommodate this complexity: an early model (with limited information available on bedside examination) guiding SE management, and a later model (incorporating a more complete clinical picture) predicting long-term functional outcome.

In this context, as displaced by Figure 1, identical input variables (such as specific EEG patterns) may carry different prognostic weight at early versus late stages. This dynamic weighting enables the model to reflect the patient’s evolving trajectory, rather than relying on a static snapshot.

## Conclusion

Prognostic SE scores represent a significant step toward structured risk stratification; however, their current utility remains limited. Most tools focus on short-term mortality, while long-term outcomes (functional recovery, epilepsy development, recurrence risk) are insufficiently addressed. Methodological constraints, reliance on subjective variables, and heterogeneity in SE definitions and treatment practices further undermine their generalizability. Artificial intelligence offers potential to overcome some of these limitations, but interpretability and data quality remain major barriers. Only if coupled with standardized definitions, EEG interpretations and harmonized care pathways, prognostic scores could gradually evolve into clinically relevant tools. A probabilistic, two-stage model that integrates early static variables with later dynamic data may represent the most appropriate strategy. Future prospective studies will be essential to establish whether they can reliably support decision-making and improve patient care.
